# Supplementary material for: Methylation profiling identifies two subclasses of squamous cell carcinoma related to distinct cells of origin
Source: Nat Commun. 2018 Feb 8;9:577. doi: 10.1038/s41467-018-03025-1 (PMC5805678; doi:10.1038/s41467-018-03025-1)
Supplement: Supplementary file 1 — Supplementary Information [file 41467_2018_3025_MOESM1_ESM.pdf]

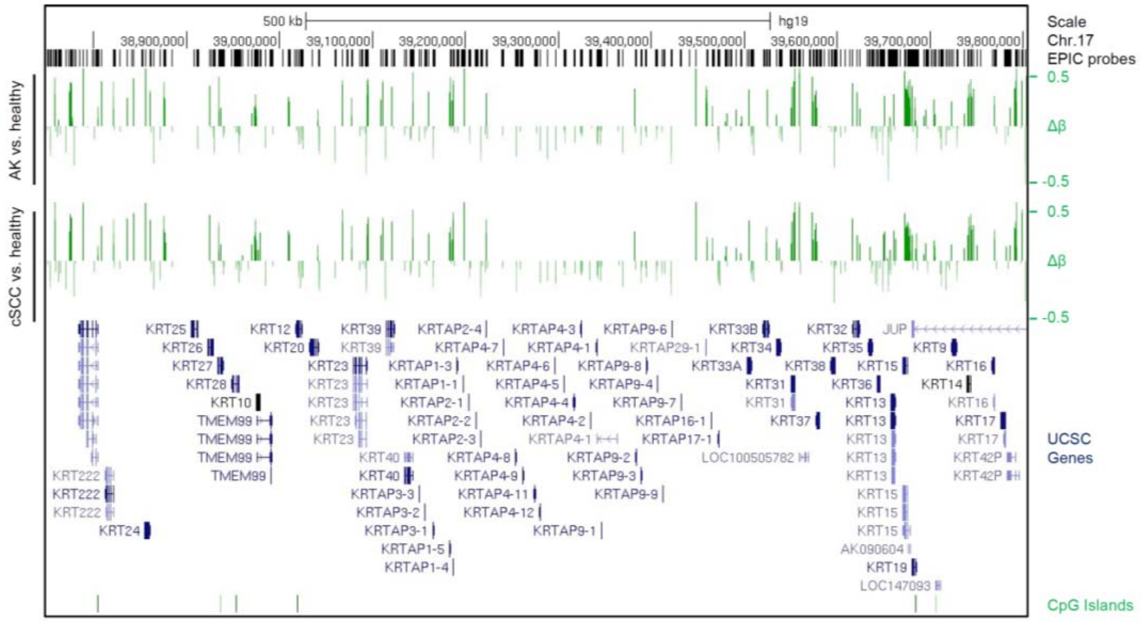

Supplementary Figure 1 University of California Santa Cruz (UCSC) genome browser tracks showing DNA methylation differences (*P*<0.05, F-test, vertical green lines) at the keratin gene cluster of chromosome 17 for Actinic keratosis (AK) (upper part) and cutaneous squamous cell carcinoma (cSCC) (lower part) in comparison to healthy epidermis.

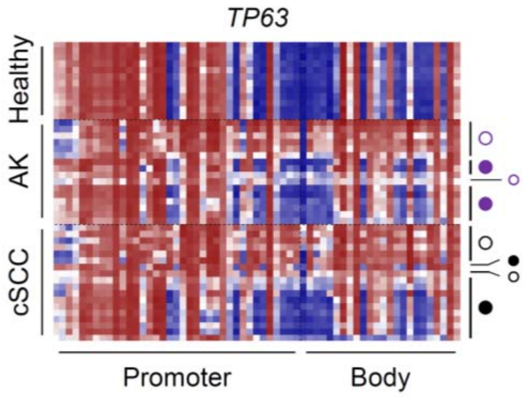

Supplementary Figure 2 DNA methylation patterns of the *TP63* gene. The heatmap shows the DNA methylation level (in β values, from blue (=0) to red (=1) of probes (columns) located in the *TP63* promoter and gene body, in healthy, AK and cSCC samples (rows). Filled and empty circles highlight the two distinct subclasses of AK (purple) and cSCC (black) samples, respectively.

IPA. Physiological System Development and Function	P-Value	Genes
Tissue development	2.92E-04-1.26E-13	407
Embryonic development	2.75E-04-2.57E-12	292
Organismal development	2.75E-04-2.57E-12	397
Cardiovascular system development and function	2.60E-04-8.19E-12	175
Nervous system development and function	2.81E-04-1.65E-11	238

Supplementary Figure 3 Ingenuity Pathway Analysis (IPA) performed with the 1188 genes located within the subset of enhancer regions lost during keratinocyte differentiation[1] and found hypermethylated in the AK/cSCC subclass related to epidermal stem cells (EpSC). *P*-values were calculated using Fisher´s exact test.

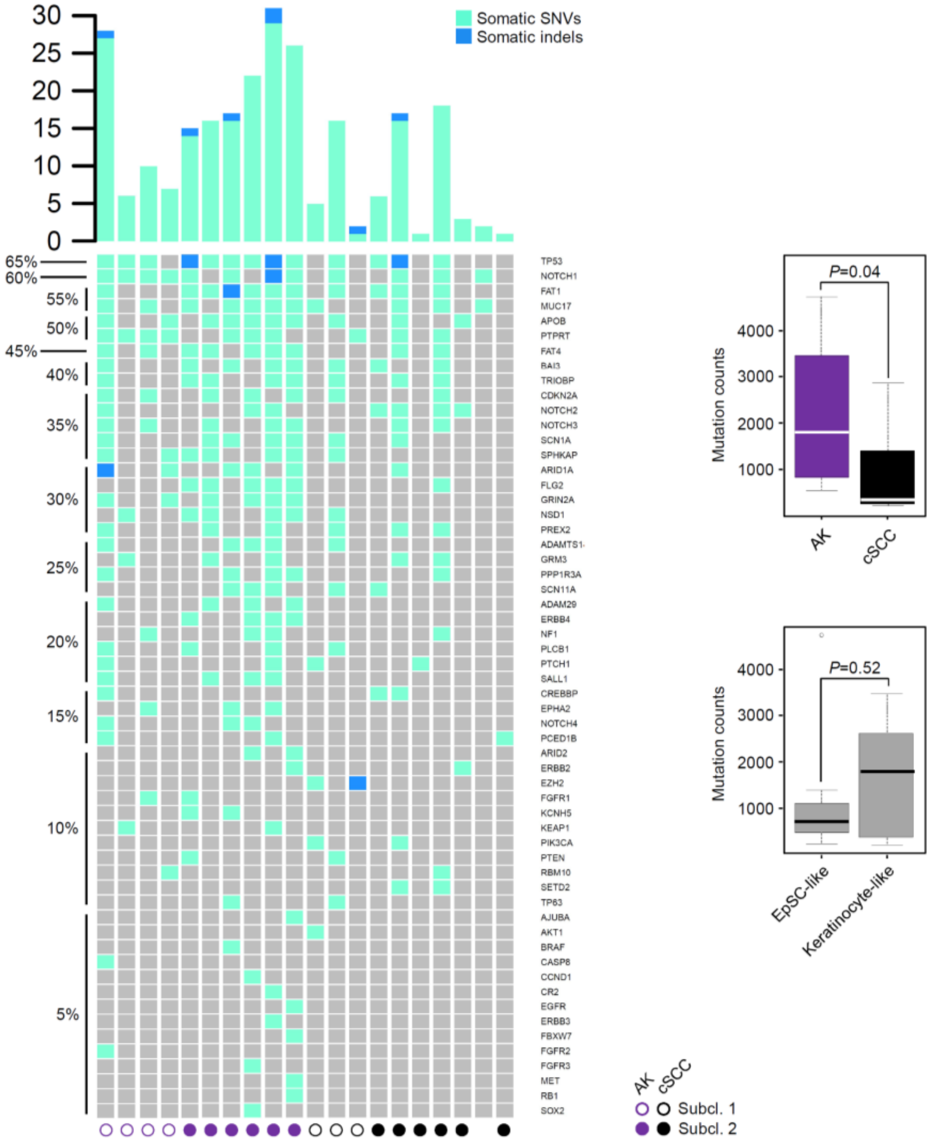

Supplementary Figure 4 Whole-exome sequencing analysis of AK and cSCC samples. The oncoprint (left) displays single nucleotide variants (green) and insertions/deletions (blue) found in each sample for the genes known to be most commonly mutated in the human epidermis[2]. Filled and empty circles indicate the two distinct subgroups of AK (purple) and cSCC (black), respectively. Box plots (right) indicate a statistically significant (*P*=0.04, two-sided t-test) higher mutation counts in AK than in cSCC (top left), but no statistically significant (*P*=0.52, two-sided t-test) differences between DNA methylation-based EpSC- and keratinocyte-like AK/cSCC subgroups.

Supplementary Table 1 Epidermis samples used in this study.

Sample ID	Status	Gender	Age	Infinium 850k	Whole-exome sequencing
HSy-01	Healthy control	Male	27	Yes	No
HSy-02	Healthy control	Male	29	Yes	No
HSy-03	Healthy control	Male	25	Yes	No
HSy-05	Healthy control	Male	19	Yes	No
HSy-06	Healthy control	Male	28	Yes	No
HSy-07	Healthy control	Male	24	Yes	No
HSo-01	Healthy control	Male	65	Yes	No
HSo-02	Healthy control	Male	73	Yes	No
HSo-03	Healthy control	Male	71	Yes	No
HSo-04	Healthy control	Male	66	Yes	No
HSo-05	Healthy control	Male	66	Yes	No
HSo-06	Healthy control	Male	67	Yes	No
AK-01	AK	Male	89	Yes	Yes
AK-03	AK	Male	85	Yes	Yes
AK-04	AK	Male	88	Yes	No
AK-05	AK	Male	76	Yes	Yes
AK-06	AK	Male	76	Yes	Yes
AK-07	AK	Male	78	Yes	Yes
AK-08	AK	Male	80	Yes	Yes
AK-09	AK	Male	84	Yes	No
AK-10	AK	Male	84	Yes	Yes
AK-11	AK	Male	83	Yes	No
AK-12	AK	Male	83	Yes	No
AK-13	AK	Female	86	Yes	Yes
AK-14	AK	Male	68	Yes	No
AK-15	AK	Male	94	Yes	Yes
AK-17	AK	Male	80	Yes	No
AK-19	AK	Male	79	Yes	Yes
SCC-02	cSCC	Male	92	Yes	Yes
SCC-03	cSCC	Male	48	Yes	No
SCC-04	cSCC	Male	85	Yes	Yes
SCC-05	cSCC	Male	79	Yes	No
SCC-06	cSCC	Male	70	Yes	Yes
SCC-07	cSCC	Male	74	Yes	Yes
SCC-08	cSCC	Male	86	Yes	Yes

SCC-09	cSCC	Male	79	Yes	Yes
SCC-10	cSCC	Male	56	Yes	No
SCC-11	cSCC	Male	92	Yes	No
SCC-12	cSCC	Male	99	Yes	No
SCC-13	cSCC	Male	88	Yes	Yes
SCC-14	cSCC	Male	68	Yes	Yes
SCC-15	cSCC	Male	78	No	Yes
SCC-16	cSCC	Male	75	Yes	No
SCC-17	cSCC	Male	88	Yes	No
SCC-18	cSCC	Male	82	Yes	Yes
SCC-19	cSCC	Male	67	Yes	No
SCC-22	cSCC	Male	103	Yes	No

Supplementary Table 2 Technical parameters of the whole-exome sequencing analysis.

Sample ID	On Target Ratio %	Coverage	Mapped reads %	Duplicates %	Properly paired %
AK-01	80.07	169.51	99.97	14.26	99.01
AK-03	81.33	160.72	99.97	19.5	99.23
AK-05	79.14	165.03	99.97	26.45	99.1
AK-06	78.99	186.58	99.97	22.19	99.17
AK-07	80.77	200.62	99.97	20.05	99.13
AK-08	79.67	205.66	99.97	19.19	99.14
AK-10	78.21	148.25	99.96	15.22	98.73
AK-13	79.31	187.09	99.97	18.93	99.13
AK-15	80.42	167.66	99.97	21.72	98.9
AK-19	80.38	178.28	99.93	19.17	98.81
SCC-02	78.99	183.76	99.97	21.72	98.91
SCC-04	79.28	175.1	99.92	19.56	98.99
SCC-06	80.74	163.87	99.98	21.49	99.06
SCC-07	78.39	188.89	99.98	19.38	99.09
SCC-08	79.2	201.05	99.98	19.56	99.08
SCC-09	79.95	191.21	99.98	21.09	98.99
SCC-13	78.75	192.96	99.97	18.34	99.08
SCC-14	80.12	172.57	99.94	19.98	98.9
SCC-15	78.3	189.12	99.95	17.49	98.76
SCC-18	80.13	178.5	99.97	19.49	98.95

Supplementary references

1. Rinaldi, L. *et al.* Dnmt3a and Dnmt3b Associate with Enhancers to Regulate Human Epidermal Stem Cell Homeostasis. *Cell Stem Cell* **19,** 491–501 (2016).

2. Martincorena, I. *et al.* Tumor evolution. High burden and pervasive positive selection of somatic mutations in normal human skin. *Science* **348,** 880–6 (2015).
